# Beware of the Pediatric Limp: A Case of *Mycoplasma* Associated Acute Transverse Myelitis

**DOI:** 10.21980/J8QQ1Q

**Published:** 2025-07-31

**Authors:** Michael Neff, Nicholas Xie, Joseph Fong, Gregory Podolej

**Affiliations:** * University of Texas Southwestern Medical Center, Department of Emergency Medicine, Dallas, TX; ^ Ascension Illinois, St. Francis Hospital, Graduate Medical Education, Evanston, IL; † University of California, San Francisco, Department of Pediatrics, San Francisco, CA; ** University of Illinois College of Medicine Peoria, Department of Emergency Medicine, Peoria, IL

## Abstract

Gait disturbances in pediatric patients can be wrought with diagnostic pitfalls given the broad infectious, traumatic, musculoskeletal, and neurological etiologies. Furthermore, children can have difficulty communicating their pain and can be hard to examine. This case highlights the importance of slowing down to obtain a detailed history as well as perform a thorough exam in our pediatric patients, which can be challenging in a busy emergency department. We present a case of acute transverse myelitis (ATM) in a four-year-old male who presented with a one-day history of limp and bilateral leg pain with weight bearing. The caretakers reported a possible remote trauma from roughhousing at daycare. Neurological examination revealed significant abnormalities including bilateral patellar hyperreflexia, bilateral lower extremity weakness, clonus, and gait ataxia. A brain and spinal MRI revealed longitudinally extensive ATM with cord edema. Despite the lack of any evidence of infectious prodrome, cerebrospinal infectious analysis revealed *Mycoplasma pneumonia* as the causative agent. Treatment with intravenous (IV) methylprednisolone and azithromycin therapy led to eventual resolution of all neurological sequelae within a few weeks of discharge. This report emphasizes the importance of considering ATM in the differential of pediatric patients presenting with gait complaints, as well as the importance of a thorough neurologic exam, without which the diagnosis would have been missed.

**Topics:**

Pediatric emergency medicine, pediatric neurology, acute transverse myelitis, pediatric gait disturbance.

**Figure:**
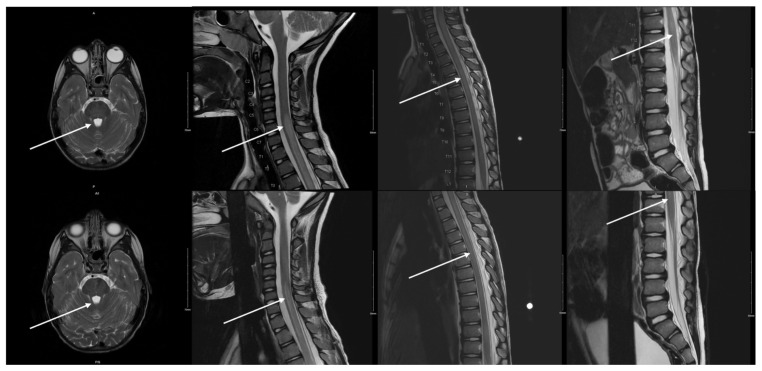


## Brief introduction

Limps account for as many as 0.7% of all pediatric emergencies.^1^ The majority of these are due to benign etiologies, and of the more serious causes, most are due to musculoskeletal infectious etiologies such as septic arthritis or osteomyelitis.^1^ Emergency physicians are well trained to consider organisms such as *Staphylococcus aureus* as a causative organism for a pediatric limp, but may not consider an atypical respiratory organism such as *Mycoplasma pneumonia* as the culprit. *Mycoplasma pneumonia* infections can have a myriad of extrapulmonary symptoms ranging from cutaneous signs to cardiovascular complications, gastrointestinal illness, and neurologic symptoms.^2^ It is rare to consider acute post-infectious neurologic etiologies in the differential of a pediatric limp, and this case report highlights the importance of a thorough exam and a broad differential.

Acute transverse myelitis (ATM) is a non-compressive, inflammatory myelopathy characterized by T2-enhancing spinal cord lesions on magnetic resonance imaging (MRI), cerebrospinal fluid (CSF) pleocytosis or increased IgG indices, and a time to maximum dysfunction between four hours and 21 days.^3^ In the United States, incidence is one to eight cases per million for all age groups, totaling approximately 1400 new cases annually. Twenty percent of cases occur in children, with a bimodal age distribution occurring between zero to two and five to 17 years.^4^ In children, acute presentations are frequently accompanied by sensory loss with a superior border above lumbar dermatomes, weakness, urinary dysfunction, pain, and inability to walk.^5^ These often present challenging diagnostic scenarios for emergency medicine (EM) physicians because presenting symptoms can be quite vague. Around 85 percent of all ATM cases are “disease-associated” with multiple sclerosis, systemic autoimmune disease, infection, radiation, drugs, toxins, and infarction as culprits.^6^ Acute transverse myelitis in children is often located centro-medullary and is longitudinally extensive (affecting three or more vertebral segments).^7^ In close to half of cases, infectious febrile illness precedes ATM symptom onset by about two to four weeks, with *Mycoplasma pneumoniae* implicated in 5-10 percent of total cases.^5^,^8^,^9^

## Presenting concerns and clinical findings

A previously healthy four-year-old boy presented to the emergency department with a one-day history of limp and trouble bearing weight on lower extremities. The patient reported a “prickly sensation” in his legs accompanied by difficulty walking and pain with weight-bearing. He had no history of recent fever or upper respiratory illness. A child had fallen onto the patient’s back at daycare two days before, resulting in a small bruise to the right hip and back pain. Physical examination on presentation was significant for bilateral, 4/5 lower extremity weakness, bilateral patellar hyperreflexia, tenderness along the midline t-spine, and clonus. He was noted to have a broad-based ataxic gait. He had an episode of urinary incontinence in the emergency room which his family noted was atypical for him. Family history was positive for Grave’s disease and Hashimoto’s thyroiditis. The constellation of these neurologic symptoms prompted the emergency physician to initiate an infectious and inflammatory workup with admission to the pediatric intensive care unit for MRIs of the brain and spine under sedation.

## Significant findings

An MRI with contrast, T2 sequence was performed. In Figures a–d, the MRI of the patient’s brain and spinal cord on admission shows abnormal signals in the patient’s pons (lack of symmetrical gray-white differentiation on cross-section) along with hyperintensity (sagittally shown as brightness in what *should* be homogenously intense spinal cord) and significant central cord edema (with swelling seen as increased width) starting from C5 and continuing to the conus medullaris around L1/L2. In Figures e-h, the MRI taken after 5 days of treatment with antibiotics and methylprednisolone shows continuing abnormal signals and hyperintensities but now with significant improvement in the quantity of cord intensity and cord width from edema.

These findings are representative of longitudinally extensive acute transverse myelitis, which is an inflammatory myelopathy that typically will present on MRI with T2-enhancing spinal cord lesions. The significant degree of edema seen on MRI did not correlate with the relatively minor severity of the patient’s physical presentation on admission. There was no concern about compressive myelopathy from neurosurgery with the imaging obtained.

## Patient course

Pediatric neurology was consulted, and initial workup showed an elevated erythrocyte sedimentation rate (ESR) at 41 millimeters per hour (mm/hr), a respiratory film array positive for rhino/enterovirus, and marked cerebrospinal fluid (CSF) pleocytosis (246 cells per cubic millimeter (cells/mm^3^). Magnetic resonance imaging of brain and spine was significant for diffuse central cord edema from C5 to conus medullaris at L1–L2 with T2 hyperintensities in the brainstem. Differentials post-MRI included compression secondary to trauma, infectious causes, autoimmune causes, and toxin exposure. Further laboratory analysis returned positive for Mycoplasma IgM in CSF as well as Mycoplasma IgG and IgM from blood samples.

The patient was started on ceftriaxone (100 mg/kg every 12 hrs) for empiric meningitis coverage initially as well as methylprednisolone (20 mg/kg for 5 days) for inflammation reduction along with famotidine (0.5 mg/kg twice daily) for stress ulcer prophylaxis. On day two, ceftriaxone coverage was switched to azithromycin (10mg/kg three times daily for 3 weeks) per Infectious Disease recommendations for treatment of suspected *M. pneumoniae* based on the CSF labs above. He remained on methylprednisolone and azithromycin until discharge on day five.

The most severe symptoms presented hours after admission, with the patient becoming incontinent, entirely unable to bear weight on the lower extremities, and developing significant thoracic spine tenderness. The patient began showing improvement in symptoms after the second dose of the methylprednisolone. His gait improved significantly, and urinary incontinence ceased after the third dose. On discharge, his physical exam had returned to baseline. Repeat MRI brain/C/T/L-spine after the five-day steroid course showed improvement in edema and in enhancement of the lesions noted in the brainstem and cervical/thoracic spine. He was sent home on continued azithromycin coverage and an extended oral prednisone taper (80 mg BID x7 days, 40 mg BID x7 days, 20 mg BID x7 days, 10 mg BID x7 days, 10 mg daily x7 days) per pediatric neurology recommendations for continued treatment of spinal cord edema.

Follow-up over the next several months outpatient showed complete resolution of all neurological sequelae. The degree and rate of this patient’s recovery are quite remarkable given the patient’s nadir and the extent of spinal and brainstem involvement.^10^

## Discussion

Acute transverse myelitis is a rare, but potentially debilitating sequelae of *M. pneumoniae* infection, especially in the pediatric population. Prompt recognition minimizes delays in the initiation of first-line, high-dose corticosteroid treatment, which has been shown to help prevent the development of permanent neurological impairment when initiated early, though a firm timeline for initiation has not been described in the literature 11. While high-dose steroids are the cornerstone of initial therapy, treatment may also include adjunctive plasmapheresis and IV immunoglobulin (IVIG), particularly in severe or refractory cases.^4^

Transverse myelitis from any cause is split into three phases: initial phase, plateau phase, and recovery phase.^7^ The initial phase is generally characterized by pain with worsening of neurological symptoms towards maximal deficit. Once at maximal deficit, plateau phase starts, where characteristic symptoms include motor, sensory, and sphincter dysfunction. Around 40 percent of pediatric ATM patients achieve full recovery, with more favorable outcomes associated with short plateau phases, the use of intravenous (IV) methylprednisolone, and plasma exchange therapy. Poor prognostic indicators include age less than or equal to three years old, diagnosis greater than or equal to seven days after symptom onset, elevated white blood cells (WBCs) in CSF, presence of T1-hypointensities on MRI, greater length of spinal cord involvement, complete paraplegia, and time to maximal deficit less than 24 hours.^5^,^10^,^12^

While the general diagnostic approach for ATM is well-defined, determining the underlying etiology can remain elusive.^6^ Imaging sensitivity and specificity aren’t well-defined, but expert consensus places MRI as the gold standard for working up acute transverse myelitis and was a crucial step in the emergency department’s approach to this patient, with CT myelography as an alternative modality in cases where MRI is not feasible.^13^, ^14^ Long segment T2 hyperintensity (with or without enhancement) on brain and spinal MRI is the common imaging manifestation of transverse myelopathies; however, it is nonspecific for parainfectious acute transverse myelitis and must be correlated with corresponding CSF infectious and neurological workup.^13^ In cases of suspected ATM, imaging workup, CSF studies, and treatment with steroids should be initiated in conjunction with expert consultation because ruling out active infectious and compressive myelopathies is crucial to avoid potential harm from steroid administration.^15^ About a third of pediatric patients with ATM have a full recovery with proper treatment. Approximately 44 percent of patients are left with neurologic sequela such as gait instability or sphincter dysfunction (Defrense). Increased severity of neurologic dysfunction, rapid time to onset of symptoms, and greater spinal lesion length have both been associated with poorer outcomes in the form of residual disability.^16^

Overall, this is a strong case for demonstrating the importance of neurological examination in pediatric patients presenting with a gait abnormality. This case has limited generalizability given that transverse myelitis is rare and the presentation of our patient’s mycoplasma infection was atypical. Considering neurologic pathologies in the differential of a pediatric limp will help identify and treat these patients during their index visit to the emergency department.

## Supplementary Information




