# Supplementary figures and images for: Beware of the Pediatric Limp: A Case of Mycoplasma Associated Acute Transverse Myelitis
Source: J Educ Teach Emerg Med. 2025 Jul 31;10(3):V22–6. doi: 10.21980/J8QQ1Q (PMC12320989; doi:10.21980/J8QQ1Q)

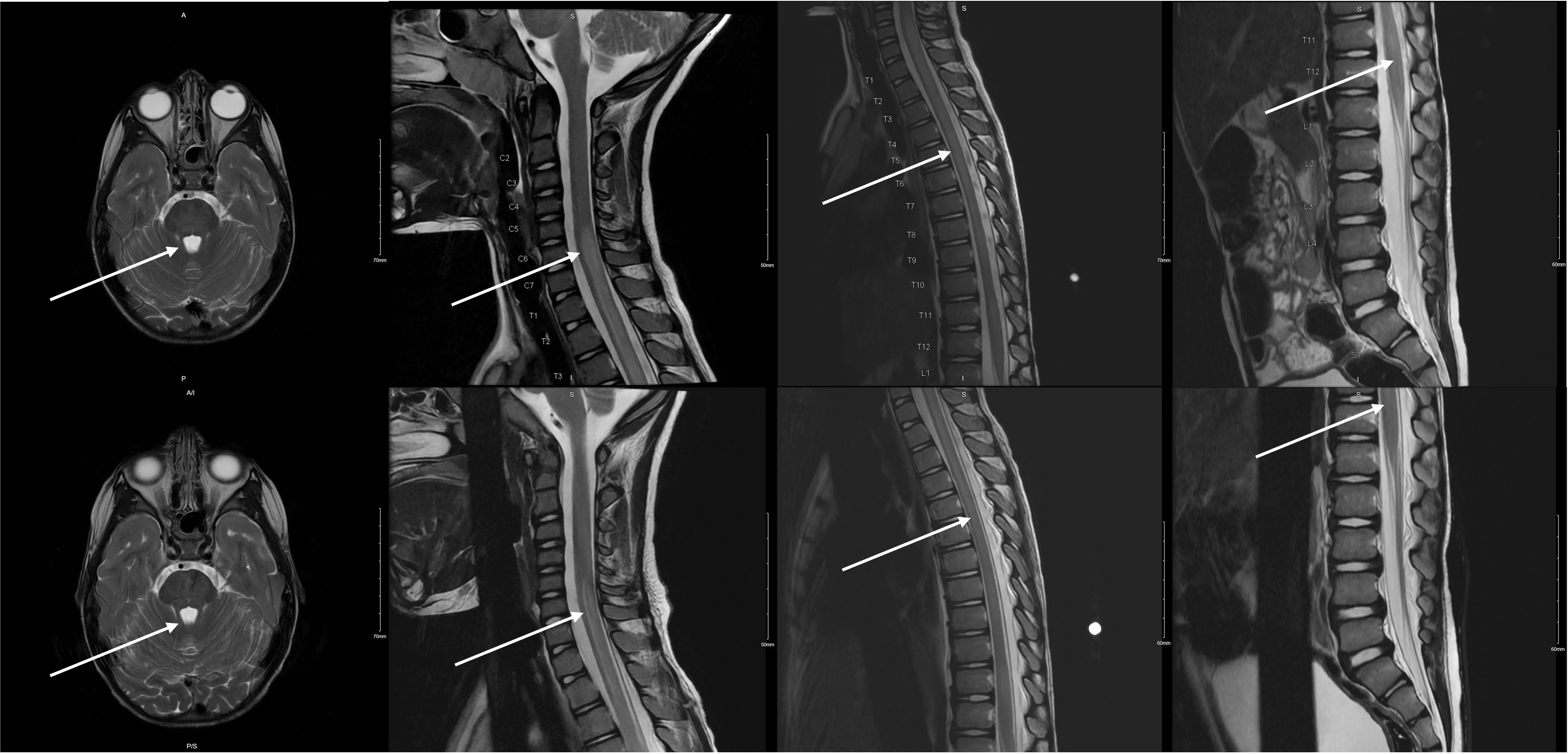

Supplement: Supplementary file 1 [file 10-3-V22-Supp1.jpg]

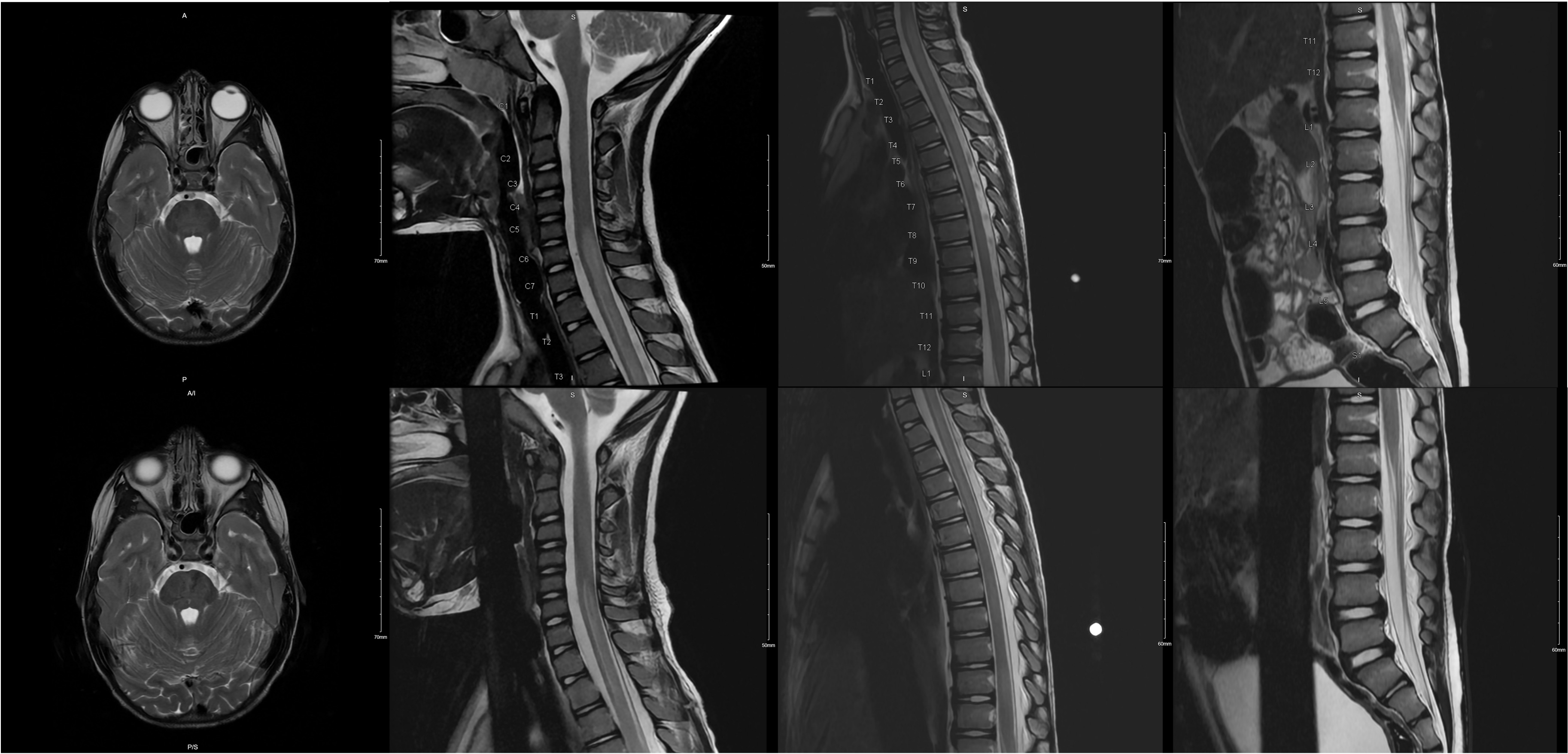

Supplement: Supplementary file 2 [file 10-3-V22-Supp2.jpg]
